# Integrated application of transcriptomics and metabolomics provides insights into gonadal differentiation in *Mesocentrotus nudus*

**DOI:** 10.1038/s41598-025-32582-x

**Published:** 2025-12-20

**Authors:** Abudula Abulizi, Weiyi Su, Xiaoxiao Huang, Heng Xiang, Zhihui Sun, Yaqing Chang

**Affiliations:** Key Laboratory of Mariculture & Stock Enhancement in North China′S Sea, Ministry of Agriculture and Rural Affairs, College of Fisheries and Life Science, Dalian Ocean University, Dalian, 116023 China

**Keywords:** Sea urchin, Gonadal differentiation, Sex-related genes, TGF-β signaling, Biochemistry, Developmental biology, Genetics, Molecular biology, Zoology

## Abstract

*Mesocentrotus nudus* is an important aquaculture species in East Asia, valued for its gonads as the only edible part. However, the molecular basis of gonadal differentiation in this species remains poorly understood. In this study, we determined that morphological gonadal differentiation occurs when individuals reach a test diameter of approximately 40 mm. Amino acid profiling revealed sex-specific differences between ovaries and testes, with higher levels of lysine, proline, alanine, and glutamic acid in testes, suggesting sexual dimorphism in metabolic demand. To investigate the regulatory mechanisms involved, we conducted integrated transcriptomic and metabolomic analyses between differentiated and undifferentiated gonads. Differentially expressed genes (DEGs) and differentially expressed metabolites (DEMs) including retinoic acid, linoleic acid, and arachidonic acid, were significantly enriched in retinol metabolism, steroid biosynthesis, and amino acid metabolic pathways. Several key genes, such as *GATA4*, *CYP17A1*, and *HSD17B*, were identified as potential markers for gonadal differentiation. Furthermore, components of the TGF-β signaling pathway (*Smads*, *Rbx1*, *SKP*) and retinol metabolism genes (*CYP26*, *CYP1A*, *CYP3A*) exhibited sex-biased expression patterns. This study provides novel insights into the molecular mechanisms underlying sex differentiation in sea urchins and lays a molecular foundation for the development of sex-control breeding strategies.

**Supplementary Information:**

The online version contains supplementary material available at 10.1038/s41598-025-32582-x.

## Introduction

Sea urchins have been widely used as model organisms in developmental biology and evolutionary research due to their unique embryology, transparent gametes, and phylogenetic position as invertebrate deuterostomes^1–4^. The sea urchin gonad is a highly prized delicacy in aquaculture, renowned for its exceptional commercial value. It is widely consumed in Japan, China, Korea, the United States, Canada, Chile, and several European countries^5^. Among the edible sea urchin species, *M. nudus* and *Strongylocentrotus intermedius* are the most widely farmed in various Asian countries, particularly in China^6,7^. Driven by rising market demand, sea urchin aquaculture in China has expanded steadily, with continuous innovation in cultivation techniques^8–11^. According to the China Fisheries Statistical Yearbook (2025), the aquaculture production of sea urchins reached approximately 7,375,843 tons^12^. Based on FAO data, global capture production of sea urchins in aquaculture was estimated to be just over 9,000 tons in 2020^13^.

The gonadal quality of sea urchins, defined by attributes such as gonadal index, plumpness, color, and appearance, is not only a key determinant in market grading and pricing but also the primary target for selective breeding programs^14^. Studies have shown that sex is a major factor influencing sea urchin quality. Comparative analyses of gonadal nutritional composition and characteristics indicate that male gonads are generally more palatable and nutritious^15–17^. However, some studies suggest that females have a higher fatty acid content and better color, which can influence market value, and reflect sex-specific metabolic demands during gonadal development^18–22^. Moreover, sex-based differences in the expression of genes related to immune and stress responses have been observed in sea urchin coelomocytes^23^. Therefore, understanding the gonadal development process and its underlying molecular mechanisms is of great importance.

Numerous studies have shown that sex determination mechanisms are highly diverse and complex, yet those in echinoderms remain unclear. In contrast to vertebrates, which typically rely on well-defined sex-determining genes (*SRY*, *DMRT1*)^24,25^ and structured somatic support cells (Sertoli and granulosa cells) within the gonads^26,27^, invertebrates such as sea urchins lack heteromorphic sex chromosomes^28,29^ and possess different gonadal architectures, including nutritive phagocytes as the primary somatic component^30,31^. Furthermore, the timing of gonadal differentiation varies substantially across taxa^32–34^ and accurately identifying this timing is essential for implementing effective sex control breeding strategies. Advances in high-throughput sequencing technology have further enabled the decoding of gene regulatory networks involved in oogenesis and spermatogenesis. Many studies have utilized transcriptome sequencing to identify genes involved in sea urchin gonadal development. For example, *S. intermedius* and *M. nudus* have been investigated using RNA-Seq to identify differentially expressed genes in the testis and ovary^35–37^, as well as sex-biased microRNAs in gonadal tissues^38^. Recent studies have revealed that sex-specific gene expression occurs prior to metamorphosis in sea urchins, indicating that sex determination may begin at the larval stage. The early activation of meiotic and sex-related genes suggests the formation of a bipotential gonad primordium accompanied by sex-biased metabolic processes^39^. In addition, multiple sex-related genes have been identified in sea urchins, including members of the *CYP* family, *Forkhead box L2* (*foxl2*), *doublesex and mab-3 related transcription factor 1* (*dmrt1*), and *nanos*^40^. However, the genetic mechanisms underlying sex determination in sea urchins remain largely unclear.

*M. nudus*, a member of the Strongylocentrotidae family, is primarily found along the coasts of northern China, northern Japan, the Korean Peninsula, and the Russian Far East. To date, its genomic data remain unavailable. In the present study, we first determined the timing of morphological gonadal differentiation in *M. nudus* and analyzed the differences in amino acid composition between the testes and ovaries. Then, we applied a metabolomic approach using liquid chromatography-quadrupole time-of-flight (LC-QTOF) along with transcriptomic analysis to identify differences in metabolites and gene expression between differentiated and undifferentiated ovaries and testes, and to explore potential molecular markers for detecting gonadal differentiation status. Furthermore, linkage networks were developed by analyzing the correlations between metabolites and regulatory genes involved in the differentiation of ovaries and testes. The findings of this study will offer new insights into the molecular mechanisms underlying sex differentiation and development in sea urchins.

## Results

### Characteristics of the gonads of *M. nudus*

**Fig. 1:**
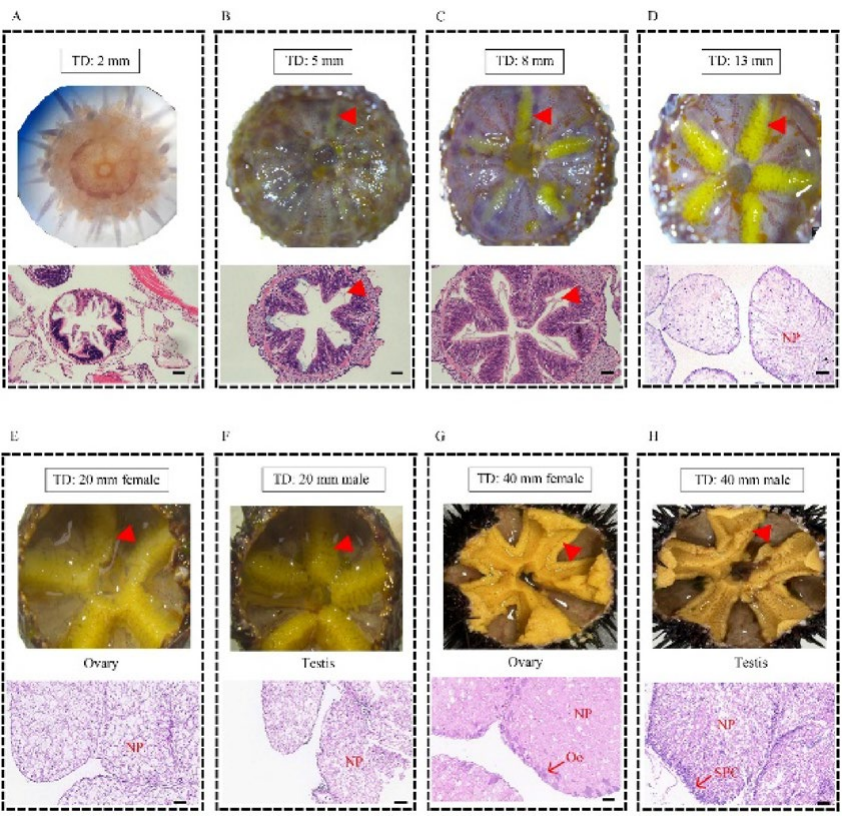
Morphological and histological observations of juvenile gonads in sea urchins. Morphological observations based on anatomical dissection (top panels) and paraffin sections of gonads (bottom panels). Red triangles indicate the gonads. (**A**–**D**) represent *M. nudus* individuals with test diameters (TD) of 2, 5, 8, and 13 mm, respectively. (**E**, **F**) represent female and male sea urchins with a TD of 24 mm, and (**G**, **H**) represent female and male sea urchins with a TD of 40 mm. TD, test diameter; NP, nutritive phagocytes; Oo, oogonia; SPC, spermatocyte. Bar = 50 µm.

Given that sea urchins attain sexual maturity at 2–3 years of age, we examined gonadal development from 2 months after hatching (test diameter ~ 2.0 mm) to 2 years of age (test diameter ~ 40 mm). Firstly, the genetic sex of sea urchins was determined by PCR amplification, as described in our laboratory’s previous report^41^. As shown in Fig. 1, gonadal tissue becomes microscopically detectable within the coelom of sea urchins once the test diameter reaches ~ 5 mm (Fig. 1A, B). As individuals grow (test diameter ~ 8.0–13 mm), the gonadal tissue within the coelom progressively expands (Fig. 1 C, D). At this stage, the gonads of sea urchins remain undifferentiated, with sexually indistinguishable gonial cells lining the inner wall. By 2 years of age (test diameter ~ 40 mm), gonads begin to differentiate, with primary oocytes and basophilic spermatogonia or primary spermatocytes appearing in the germinal epithelium of ovaries and testes, respectively (Fig. 1 E–H). Moreover, differentiated testes exhibited higher proportions of lysine (an essential amino acid) and certain nonessential amino acids, including glutamic acid, glycine, alanine, and proline, compared to differentiated ovaries (Table 1). In contrast, no significant differences were observed in the fatty acid composition among the samples analyzed (Table S1).

**Table 1:** Amino acid composition analysis of gonadal tissues in adult sea urchins.

Essential amino acids	Ovary (%)	Testis (%)	*p-value*	Nonessential amino acids	Ovary (%)	Testis (%)	*p-value*
Leucine	7.35 (0.08)	8.00 (0.07)	0.60	Glutamic acid	9.10 (0.10)	13.70 (0.12)	0.01
Lysine	6.77 (0.08)	10.78 (0.09)	0.03	Glycine	8.95 (0.10)	11.48 (0.10)	0.05
Valine	6.25 (0.07)	6.14 (0.05)	0.91	Arginine	7.37 (0.08)	12.48 (0.11)	0.15
Threonine	5.21 (0.06)	5.68 (0.05)	0.64	Aspartic acid	7.62 (0.09)	10.99 (0.10)	0.07
Isoleucine	4.88 (0.06)	5.24 (0.05)	0.67	Serine	5.00 (0.06)	6.02 (0.05)	0.21
Phenylalanine	4.32 (0.05)	5.12 (0.04)	0.31	Tyrosine	4.60 (0.05)	4.54 (0.04)	0.95
Methionine	2.01 (0.02)	2.37 (0.02)	0.57	Alanine	3.98 (0.04)	5.60 (0.05)	0.01
Histidine	2.18 (0.02)	2.56 (0.02)	0.38	Proline	2.97 (0.03)	4.38 (0.04)	0.04

Data are presented as mean values (g/kg), with percentages (%) indicating relative proportions of total amino acids. *P* < 0.05 was considered statistically significant.

### Transcriptome analysis of undifferentiated and differentiated gonads

#### Overview of transcriptome sequencing

Total RNA was extracted from differentiated ovaries and testes (test diameter ~ 40 mm) and undifferentiated ovaries and testes (test diameter ~ 20 mm) for transcriptome sequencing. A total of 277,860,486 clean reads were generated, with a mean GC content of 43.2% and a mean Q30 of 95.25% (Table S2). Through de novo assembly, a total of 56,770 unigenes were obtained with an N50 value of 2,846 (Table S3). Subsequently, 25,652 unigenes were annotated by aligning them with various databases, including NR, SWISSPROT, COG, KOG, EGGNOG, KEGG, GO, PFAM and TREMBL database (Table S4). Principal component analysis (PCA) showed clear separation among the four groups: differentiated ovaries (DO), differentiated testes (DT), undifferentiated ovaries (UDO), and undifferentiated testes (UDT) (Figure S1). A total of 5,085 differentially expressed genes (DEGs) were identified between the DO and DT groups (fold change ≥ 2 and *p* < 0.01). Among these, 2,641 were up-regulated and 2,444 were down-regulated in the DT populations. Compared to the UDO sea urchins, the UDT group had 374 upregulated genes and 620 downregulated genes. In addition, compared with the differentiated ovaries and testes, the undifferentiated ovaries and testes showed 2,011 and 3,977 upregulated genes, and 2,718 and 4,185 downregulated genes, respectively (Figure S2).

#### DEGs enrichment results in GO and KEGG

**Fig. 2:**
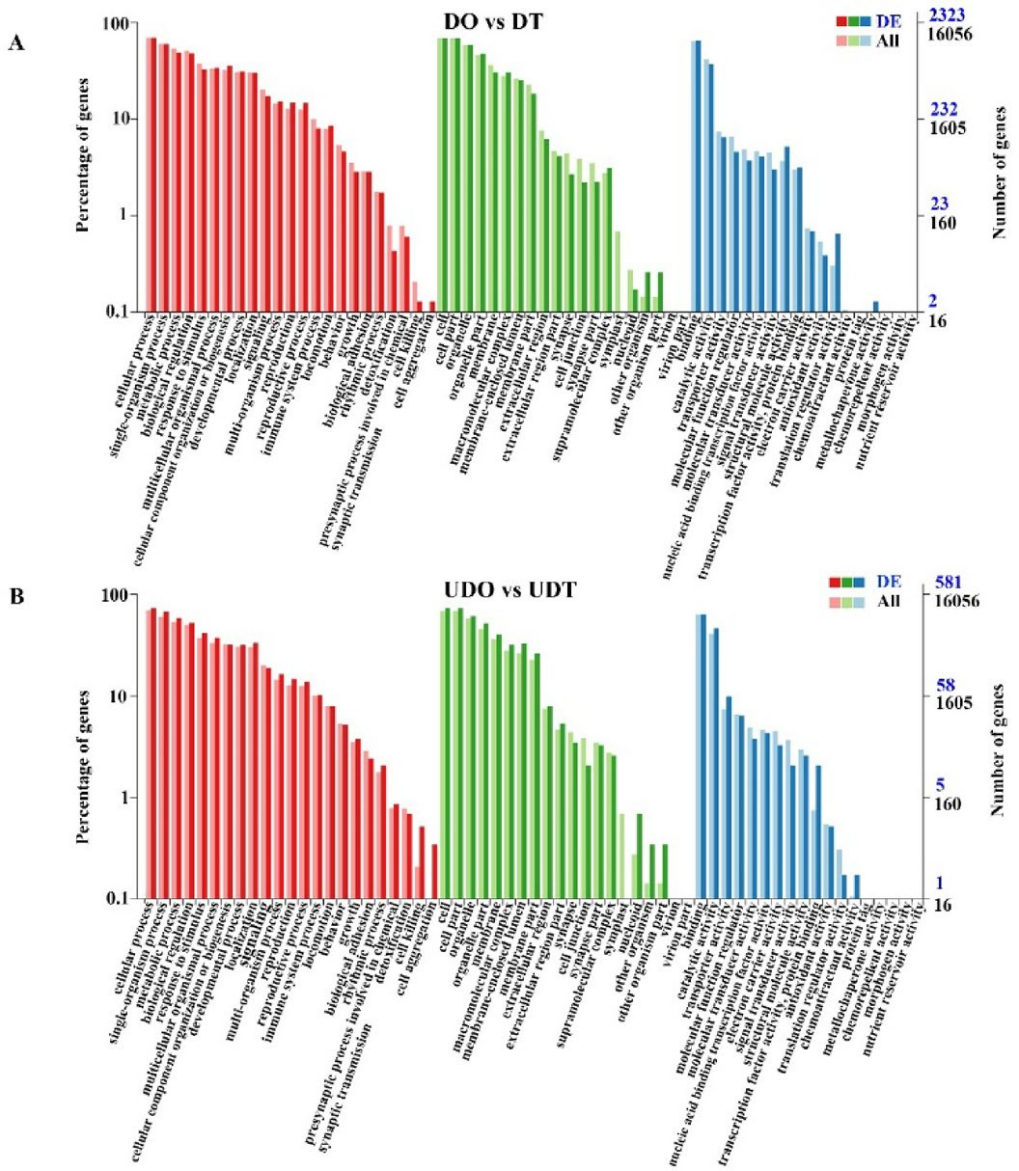
GO term enrichment analysis was performed for the DEGs between the compared gonadal groups. (**A**) GO term enrichment analysis of DEGs between differentiated ovaries and testes. (**B**) GO term enrichment analysis of DEGs between undifferentiated ovaries and testes.

**Fig. 3:**
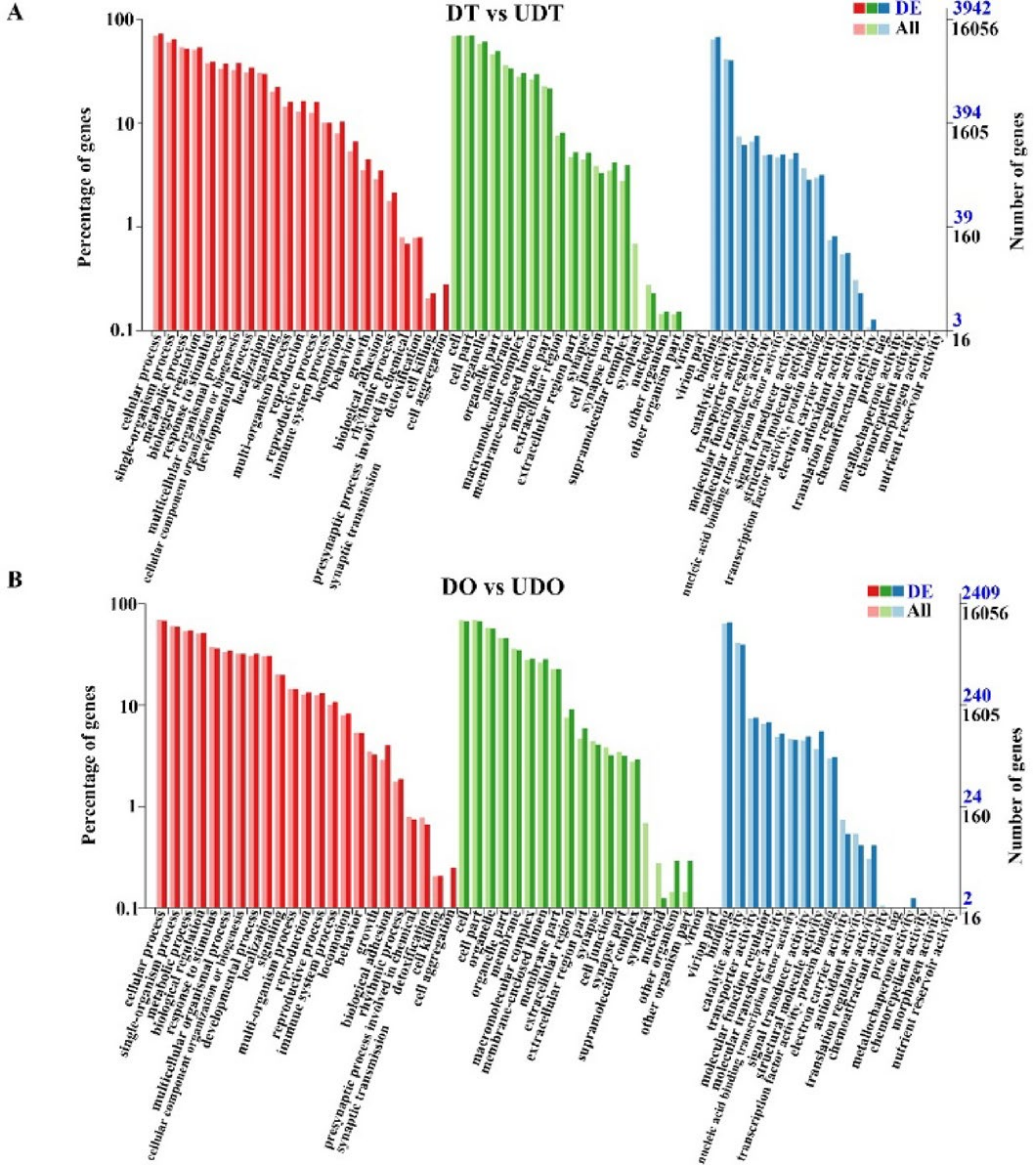
GO term enrichment analysis was performed for the DEGs between the compared gonadal groups. (**A**) GO term enrichment analysis of DEGs between differentiated testes and undifferentiated testes. (**B**) GO term enrichment analysis of DEGs between differentiated ovaries and undifferentiated ovaries.

To further determine the DEGs functions, we mapped all DEGs to GO and KEGG pathways. Compared to the DO group, the DT group displayed the most significantly enriched GO terms related to biological processes, including cellular component organization or biogenesis, reproductive process and reproduction. Enriched molecular function terms included structural molecule activity, translation regulator activity, and binding, while macromolecular complex, host cell part, and organelle part were the most enriched terms among cellular components (Fig. 2A). Similarly, compared to the UDO group, the UDT group exhibited highly enriched GO terms related to biological processes, such as metabolic process, cellular process, and response to stimulus, along with enriched molecular function terms catalytic activity, electron carrier activity, and binding, and cellular component terms membrane-enclosed lumen, nucleoid, and organelle part (Fig. 2B). In comparison to the DT group, the UDT group displayed enrichment in biological process terms cellular component organization or biogenesis, reproduction, and reproductive process, molecular function terms binding, molecular function regulator, and molecular transducer activity, and cellular component terms membrane-enclosed lumen, organelle part, and supramolecular complex (Fig. 3A). Lastly, the UDO group, when compared to the DO group, exhibited enriched GO terms for biological processes such as biological adhesion, metabolic process, and developmental process, molecular functions structural molecule activity, molecular transducer activity, and translation regulator activity, and cellular components extracellular region part, extracellular region, and membrane-enclosed lumen (Fig. 3B).

**Fig. 4:**
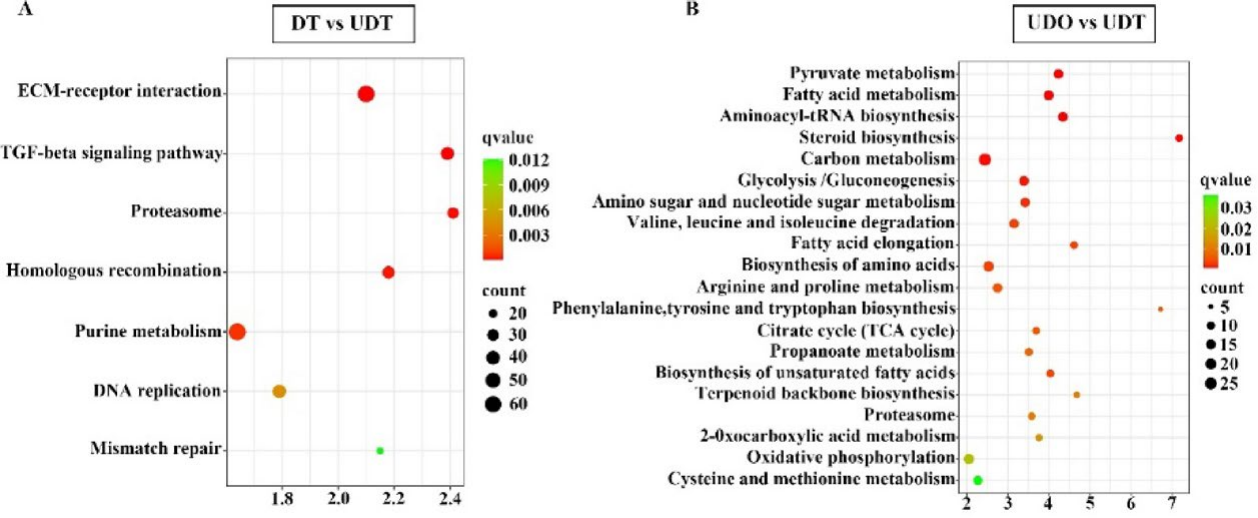
KEGG pathway analysis of DEGs between the compared gonadal groups. (**A**) KEGG pathway analysis of DEGs between differentiated testes and undifferentiated testes. (**B**) KEGG pathway analysis of DEGs between undifferentiated ovaries and undifferentiated testes. The vertical axis shows KEGG pathways that are significantly enriched by DEGs, and the horizontal axis indicates the corresponding enrichment factors. Pathway maps are based on the KEGG database ^42–44^.

KEGG enrichment analysis identified significantly altered pathways (*q* < 0.05) in the transcriptome, with slight variations observed among the four groups. Specifically, in the DO vs. DT comparison, two metabolic pathways, ribosome (82 DEGs; upregulated: 3, downregulated: 79) and purine metabolism (40 DEGs; upregulated: 25, downregulated: 15) exhibited significant changes. In the DO vs. UDO comparison, 1,149 genes were mapped to 231 metabolic pathways, with only the ribosome pathway (81 DEGs; upregulated: 1, downregulated: 80) showing significant enrichment. In the DT vs. UDT comparison, seven pathways showed significant changes: extracellular matrix-receptor interaction (62 DEGs; upregulated: 55, downregulated: 7), TGF-β signaling pathway (36 DEGs; upregulated: 30, downregulated: 6), proteasome pathway (28 DEGs; upregulated: 1, downregulated: 27), homologous recombination (32 DEGs; upregulated: 1, downregulated: 31), purine metabolism (64 DEGs; upregulated: 23, downregulated: 41), DNA replication (37 DEGs; upregulated: 5, downregulated: 32), and mismatch repair (19 DEGs; upregulated: 2, downregulated: 17) (Fig. 4A). Finally, in the UDO vs. UDT comparison, 326 genes were mapped to 135 metabolic pathways, with 22 pathways exhibiting significant changes (Fig. 4B).

#### Identification of key sex-biased genes

Based on gene functions annotated by GO and KEGG, we identified several conserved sex-related genes that exhibited significant expression changes before and after gonadal differentiation, including *hydroxysteroid 17-beta dehydrogenase* (*HSD17B*), *GATA binding protein 4* (*GATA4*), *SRY-box transcription factor 14* (*SOX14*), *cytochrome P450 family 17 subfamily A member 1* (*CYP17A1*), *CYCLIN A and CYCLIN B*, *DMRT1*, *FOXL2*, *SIX homeobox 4* (*SIX4*), *ACTIVIN*, *Wnt inhibitory factor-1* (*WIF1*). Based on the FPKM values from transcriptome data (Table S5), we found that *GATA4*, *CYP17A1*, *FOXL2*, *ACTIVIN* and *WIF1* were predominantly expressed in undifferentiated gonads, suggesting their potential roles in the early stages of gonadal formation. In contrast, *SOX14*, *HSD17B*, *CYCLIN A*, and *CYCLIN B* showed higher expression in differentiated gonads, with *SOX14* and *DMRT1* exhibiting the highest expression levels in differentiated testes (Table 2).

**Table 2:** Information on sex-biased gene expression between differentiated and undifferentiated gonads of *M. nudus*.

Gene name	Function	Expression level			
		DO	DT	UDO	UDT
*SOX14*	Involved in sex determination and differentiation, gonadal development, neurogenesis, early embryonic development^45^	1.66^a^	39.55^a/c^	1.04^d^	4.37^c/d^
*HSD17B*	Regulating transformation among androstenedione, testosterone, estrone and estradiol^46^	14.78^a/b^	10.44^a/c^	4.05^b^	2.74^c^
*GATA4*	Regulating the proliferation, differentiation and apoptosis of ovarian granulosa cells, promoting spermatogenesis and sex differentiation^47^	16.86^b^	17.69^c^	46.38^b^	50.6^c^
*CYP17A1*	Catalyzes steroid hormone synthesis, essential for androgen and estrogen production and gonadal development^48^	2.47^b^	2.28^c^	5.19^b^	6.89^c^
*CYCLIN A*	Regulates DNA replication, mitosis, and meiosis^49^	73.34^b^	46.86^c^	1.89^b^	2.45^c^
*CYCLIN B*	Regulates cell proliferation and promotes gonad development, mitosis, and meiosis^50^	113.06^b^	97.94^c^	3.62^b^	3.58^c^
*DMRT1*	Regulates sex determination and sex differentiation, testis development and male sex differentiation, important for spermatogenesis^51^	0^a^	22.24^a/c/d^	0	1.96^c/d^
*FOXL2*	Regulates ovarian development and maintenance, involved in granulosa cell differentiation^52^	6.23^b^	7.18^c^	22.38^b^	33.35^c^
*SIX4*	Involved in gonad and muscle development; regulates cell differentiation and organogenesis^53^	1.29	2.65^c^	0.77^d^	0.52^c/d^
*ACTIVIN*	Regulates embryonic development, skeletal muscle, and reproductive development^54^	1.02^b^	2.67	4.24^b^	4.45
*WIF1*	Inhibits Wnt signaling; involved in cell differentiation and tissue development^55^	2.78	1.5^c^	5.89	6.25^c^

DO, differentiated ovaries; DT, differentiated testes; UDO, undifferentiated ovaries; UDT, undifferentiated testes. Different letters indicate statistically significant differences between groups (*p* < 0.05): a, DO vs. DT; b, DO vs. UDO; c, DT vs. UDT; d, UDT vs. UDO.

**Fig. 5:**
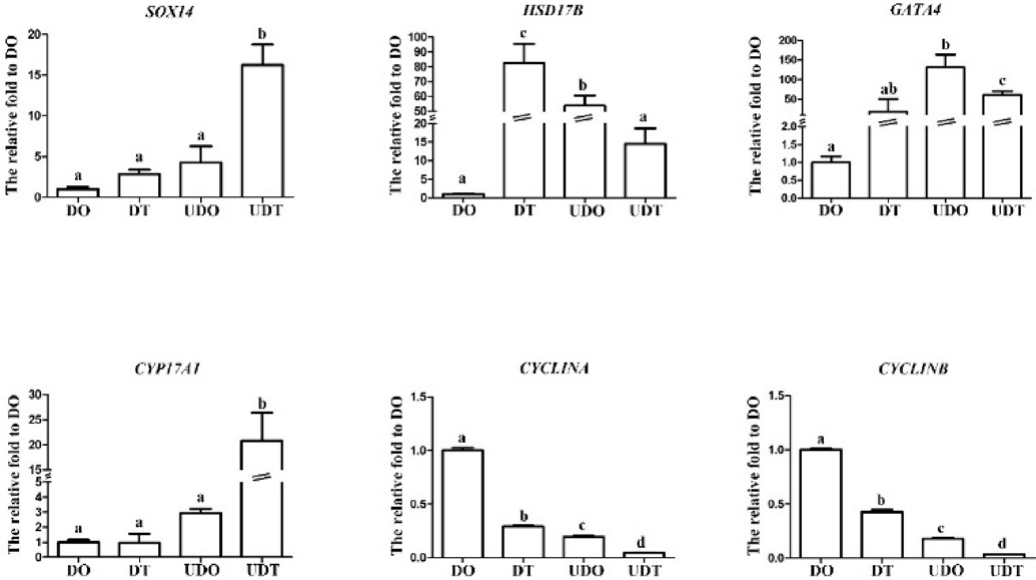
Validation of six key differentially expressed genes in *M. nudus* by qRT-PCR. Data are expressed as the mean ± standard deviation (SD) of three replicates. DO, differentiated ovaries; DT, differentiated testes; UDO, undifferentiated ovaries; UDT, undifferentiated testes. Identical letters indicate no significant differences among individuals, whereas different letters denote significant differences (*p* < 0.05).

To validate the expression patterns of DEGs identified through Illumina sequencing, several key differential genes were further analyzed using qRT-PCR. The qRT-PCR results confirmed that their expression levels were consistent with the RNA-seq findings (Fig. 5). These results indicate that the observed changes in gene expression detected by RNA-seq accurately reflect the actual transcriptomic differences among the gonadal libraries.

### Metabolomics analysis of undifferentiated and differentiated gonads

#### Metabolite differences between undifferentiated and differentiated gonads

To explore the metabolic differences among DO, UDO, DT, and UDO gonads in sea urchins, we generated comprehensive profiles of primary metabolites using UPLC-MS/MS. A total of 4,827 metabolites were identified across the four gonadal types. Among these, 975 metabolites showed significant changes between the DO and DT groups, with 481 upregulated and 494 downregulated (Figure S3). In the DO vs. UDO comparison, 2,520 differential metabolites were identified (1,164 upregulated and 1,356 downregulated), while in the DT vs. UDT comparison, 2,452 differential metabolites were observed (982 upregulated and 1,470 downregulated). Meanwhile, compared to the UDO group, 799 differential metabolites were identified in the UDT group, comprising 392 upregulated and 407 downregulated. A total of 56 differential metabolites were shared among the four comparison groups. The differentially expressed metabolites (DEMs) primarily between the DO and DT groups mainly consisted of Pneumocandin B0, Luteolin 7-O-(2-apiosyl-4-glucosyl-6-malonyl)-glucoside, purine nucleosides, PA(LTE4/18:3(9Z,12Z,15Z)), Zotiraciclib and L-Tryptophan, N-(N(2)-(N-(N-(N-L-methionyl-L-alpha-glutamyl)-L-histidyl)-L-phenylalanyl)-L-arginyl). The DEMs predominantly consist of Thioridazine, Buprenorphine, Notoginsenoside I, PA(LTE4/18:3(9Z,12Z,15Z)), Zotiraciclib, and Cyclosquamosin D between the UDO and UDT groups. Meanwhile, Anabasine, Torulene, Protionamide sulfate, Methyl (Z)-5-(5-methyl-2-thienyl)-2-penten-4-ynoate, Furamidine, and 7-Mercaptoheptanoylthreonine are the main DEMs in the DO and UDO groups, and PIP(22:3(10Z,13Z,16Z)/20:4(5Z,8Z,11Z,14Z)-OH(20)), Elaterinide, CDP-DG(18:2(9Z,11Z)/PGE2), Pneumocandin B0, Methionyl-Arginine, and t Kaempferol 3-rhamnosyl-(1- > 3)(4'''-acetylrhamnosyl)(1- > 6)-glucoside are the main DEMs in the DT and UDT groups (Table 3).

**Table 3:** Top three upregulated and downregulated metabolites.

Group	DEMs ID	Name	Log_2_ FC	*p-value*	Regulated
DO vs DT	pos_7648	Pneumocandin B0	37.64	0.03	up
	pos_3749	Luteolin 7-O-(2-apiosyl-4-glucosyl-6-malonyl)-glucoside	36.67	0.04	up
	pos_2057	methionine aspartate aspartate	36.59	0.04	up
	pos_4788	PA(LTE4/18:3(9Z,12Z,15Z))	-43.31	0.00	down
	neg_2555	Zotiraciclib	-42.06	0.00	down
	neg_2342	L-Tryptophan, N-(N(2)-(N-(N-(N-L-methionyl-L-alpha-glutamyl)-L-histidyl)-L-phenylalanyl)-L-arginyl)-	-40.97	0.01	down
DO vs UDO	neg_4104	Thioridazine	36.60	0.00	up
	neg_3564	Buprenorphine	35.73	0.02	up
	pos_8367	Notoginsenoside I	35.65	0.01	up
	pos_4788	PA(LTE4/18:3(9Z,12Z,15Z))	-43.31	0.00	down
	neg_2555	Zotiraciclib	-42.06	0.00	down
	neg_2530	Cyclosquamosin D	-41.67	0.01	down
DT vs UDT	pos_7729	PIP(22:3(10Z,13Z,16Z)/20:4(5Z,8Z,11Z,14Z)-OH(20))	36.59	0.02	up
	neg_3678	Elaterinide	35.39	0.01	up
	neg_3291	CDP-DG(18:2(9Z,11Z)/PGE2)	35.03	0.00	up
	pos_7648	Pneumocandin B0	-37.64	0.04	down
	pos_967	Methionyl-Arginine	-36.14	0.02	down
	pos_1925	Kaempferol 3-rhamnosyl-(1- > 3)(4'''-acetylrhamnosyl)(1- > 6)-glucoside	-34.85	0.05	down
UDO vs UDT	pos_7367	Anabasine	4.97	0.04	up
	neg_1699	Torulene	4.83	0.03	up
	pos_4721	Protionamide sulfate	4.39	0.05	up
	pos_983	Methyl (Z)-5-(5-methyl-2-thienyl)-2-penten-4-ynoate	-5.32	0.04	down
	pos_10052	Furamidine	-4.03	0.05	down
	pos_1872	7-Mercaptoheptanoylthreonine	-3.92	0.04	down

#### KEGG analysis of differential metabolites

**Fig. 6:**
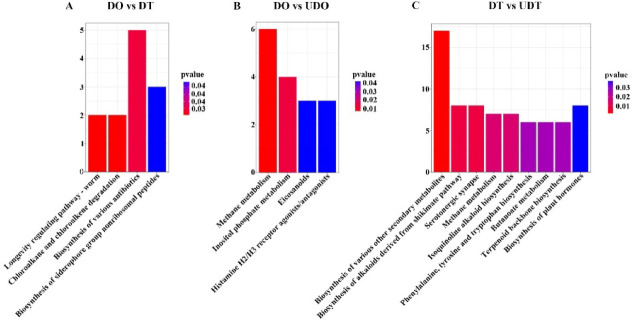
KEGG pathway analysis of DEMs between the compared gonadal groups. The vertical axis represents the number of DEMs associated with each pathway, while the horizontal axis indicates the KEGG significantly pathways enriched by DEMs. Pathway maps are based on the KEGG database ^42–44^.

In the DO vs. DT group, 80 differential metabolites were enriched in KO pathways, with the most significant pathways including longevity regulating pathway—worm, the chloroalkane and chloroalkene degradation, the biosynthesis of various antibiotics, and the biosynthesis of siderophore group nonribosomal peptides (Fig. 6A). In the DO vs. UDO group, 229 differential metabolites were enriched in KO pathways, primarily associated with methane metabolism, inositol phosphate metabolism, eicosanoids, and histamine H2/H3 receptor agonists/antagonists (Fig. 6B). In the DT vs. UDT group, 236 differential metabolites were enriched in KO pathways, with key pathways including the biosynthesis of alkaloids derived from the shikimate pathway, the biosynthesis of various other secondary metabolites, serotonergic synapse, methane metabolism, isoquinoline alkaloid biosynthesis, phenylalanine, tyrosine and tryptophan biosynthesis, butanoate metabolism, terpenoid backbone biosynthesis, and biosynthesis of plant hormones. (Fig. 6C) In the UDO vs. UDT group, 75 differential metabolites were enriched in KO pathways, mainly related to penicillins and the biosynthesis of siderophore group nonribosomal peptides.

### Integrative analysis of metabolomics and transcriptomics

**Fig. 7:**
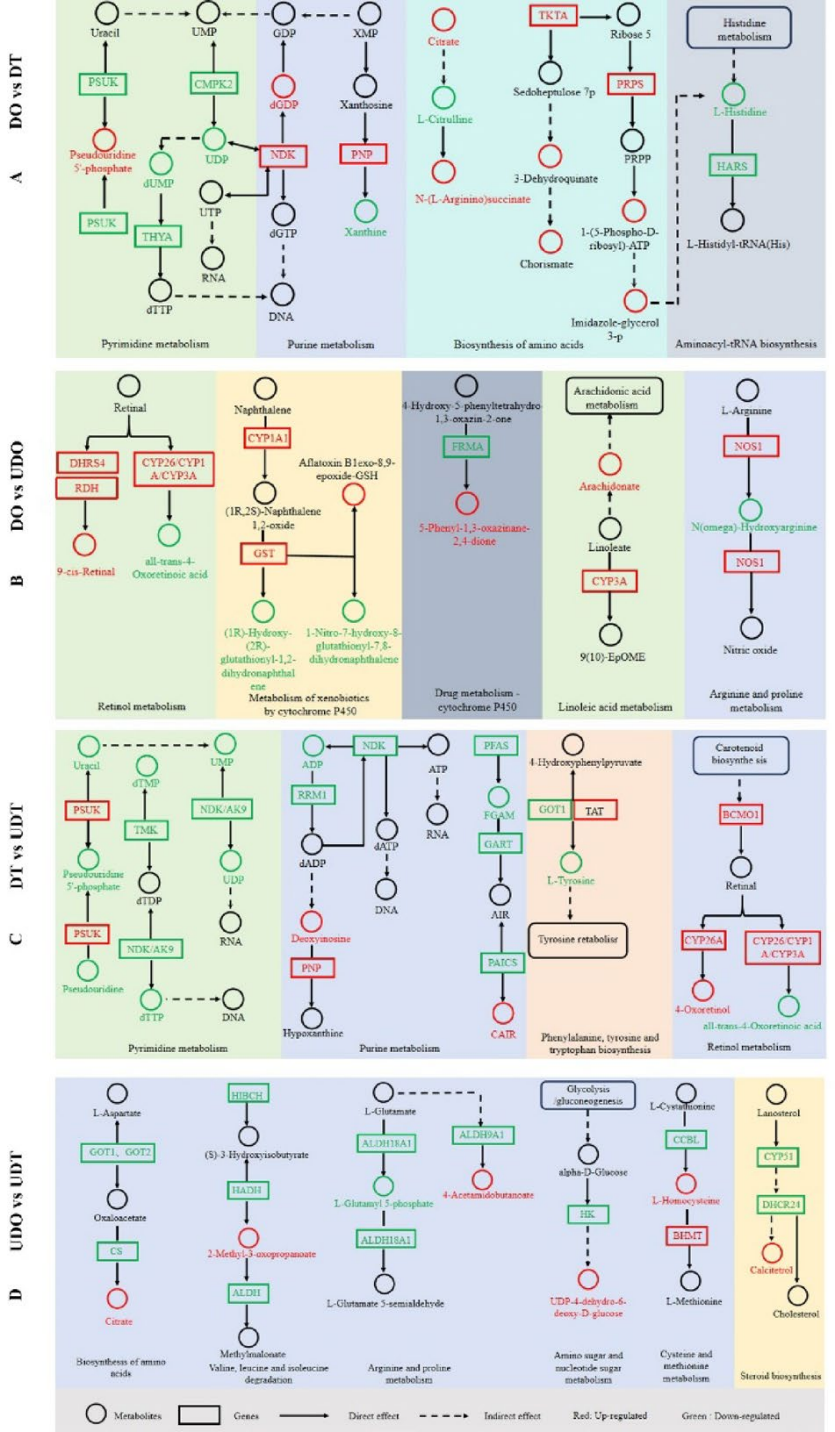
An integrative metabolic network map constructed from differentially expressed genes and metabolites between the compared gonadal groups. (**A**) DO vs DT group. (**B**) DO vs UDO group. (**C**) DT vs UDT group. (**D**) UDO vs UDT group. DO, differentiated ovaries; DT, differentiated testes; UDO, undifferentiated ovaries; UDT, undifferentiated testes. Differentially expressed genes (DEGs) are represented by boxes, and differentially expressed metabolites (DEMs) are represented by circles. Red indicates upregulation, while green indicates downregulation.

Spearman correlation analysis revealed a strong correlation between DEGs and DEMs (Table S6). In the DO vs. DT comparison, purine metabolite, pyrimidine metabolite, aminoacyl-tRNA biosynthesis, and biosynthesis of amino acids were identified as significantly co-enriched pathways. Within the DT group, the metabolites abundances of UDP, dUMP, xanthine, L-citrulline, and L-histidine were lower, whereas the levels of dGDP, pseudouridine 5’-phosphate, 3-dehydroquinate, and chorismate were higher. Additionally, *NDK* gene expression was upregulated (Fig. 7A). In the DO vs. UDO comparison, five metabolic pathways were significantly co-enriched: retinol metabolism, metabolism of xenobiotics by cytochrome P450, drug metabolism (cytochrome P450), linoleic acid metabolism, and arginine and proline metabolism. In the UDO group, the metabolites abundance of all-trans-4-oxoretinoic acid was lower, whereas 9-cis-retinal was higher, and the expression of *CYP26*, *CYP1A*, *CYP3A*, *DHRS4*, and *RHD* genes was upregulated (Fig. 7B). Compared to the DT group, the UDT group exhibited metabolites with lower abundances of ADP, UMP, uracil, dTMP, UDP, dTTP, and L-tyrosine, with *NDK*, *AK9*, *TMK*, and *GART* gene expression downregulated. This suggests that *NDK*, *TMK*, and *GART* are associated with testis differentiation (Fig. 7C). Additionally, the steroid biosynthesis pathway was significantly co-enriched in both the UDO and UDT groups. Notably, the UDT group exhibited higher levels of calcitetrol, while the expression of *CYP51* and *DHCR24* was downregulated, suggesting a potential role for steroid biosynthesis in early gonadal differentiation processes (Fig. 7D).

## Discussion

In this study, we revealed that the gonads of sea urchins with a test diameter of less than 20 mm remain undifferentiated, and morphological gonadal differentiation appears to occur when the test diameter reaches between 20 and 40 mm. Following this differentiation, sea urchins enter the reproductive cycle, progressing through four distinct stages: (1) inter-gametogenesis and nutritive phagocytes (NP) phagocytosis (Stage 1), (2) pre-gametogenesis and NP renewal (Stage 2), (3) gametogenesis and NP utilization (Stage 3), and (4) the culmination of gametogenesis (Stage 4), marked by NP exhaustion and spawning^37,56^. Previous studies have reported transcriptomic differences across these stages and identified candidate genes related to growth and fatty acid biosynthesis and metabolism, particularly between Stage 1 and Stage 2^57^. In addition, integrated transcriptomic and metabolomic analyses of gonads from Stages 1 to 3 revealed six differentially accumulated metabolites associated with polyunsaturated fatty acids (PUFAs), along with multiple DEGs involved in PUFA synthesis and regulation^58^. Here, we compared the amino acid composition results with the metabolomic data to identify consistent patterns of amino acid metabolism between differentiated ovaries and testes. Most amino acids detected in the biochemical composition analysis, including L-phenylalanine, L-arginine, L-isoleucine, L-methionine, and L-threonine, were also identified in the metabolomic dataset, and their levels showed no significant differences between differentiated ovaries and testes, supporting the reliability of the metabolic profiling. However, several highly polar or easily oxidized amino acids were not detected, likely due to their low ionization efficiency or instability during LC–MS analysis.

Unlike vertebrates, which possess specialized somatic support cells such as Sertoli cells (testis) and granulosa cells (ovary) that play active roles in directing germ cell fate^59^, sea urchin gonads contain nutritive phagocytes that perform both nutritional and regulatory functions^60^. Moreover, the timing and regulation of the germ cell cycle differ substantially between vertebrates and sea urchins. In sea urchins, germ cells typically enter meiosis at later developmental stages, and gametogenesis is tightly synchronized with seasonal and environmental cues^60,61^. However, due to the asynchronous nature of gonadal development in sea urchins and the fact that germ cell meiosis occurs entirely within the body^62^, some differentiated male samples contained a small number of spermatids, as shown in histological sections (Figure S4-I). Consequently, several meiosis-related genes, including *cntd1*, *sycp1*, *ccnb3*, and *cdk1*, were also identified in this study.

It has been reported that molecular sex differentiation occurs earlier than morphological sex differentiation^63^, therefore, identifying molecular markers for gonadal differentiation is crucial. The expression of *GATA4*, and *CYP17A1* was significantly higher in undifferentiated ovaries and testes, while *HSD17B* exhibited higher expression in differentiated ovaries and testes (Fig. 5). These genes may serve as potential molecular markers for identifying gonadal differentiation status in sea urchins and are known to play roles in gonadal development across vertebrate species. In mammals, *GATA4* plays a key role in reproductive and gonadal differentiation during embryonic development; its conditional knockout in mice reduces sperm quantity and motility, causes Sertoli cell vacuolation, impairs spermatogenesis, and results in testicular atrophy and infertility^64,65^. In invertebrates, *GATA4* has been identified in *Argopecten* scallops, where its expression is higher in testes than in ovaries; however, its precise function in gonadal development remains unclear^66^. *Cyp17a1* is essential for the biosynthesis of sex steroid hormones and plays a crucial role in female sex determination in vertebrates^48^. Although homologs of *CYP17A* have been identified in various invertebrate species and exhibit sexually dimorphic expression patterns, their functional roles in gonadal development remain largely uncharacterized^67^. *HSD17B* enzymes play key roles in lipid and steroid metabolism and have been extensively studied in vertebrates^68^. In invertebrates, such as *Patinopecten yessoensis*, high expression levels of *hsd17b8* and *hsd17b11* were observed in ovaries, and their expression was associated with gonadal maturation, showing a progressive increase from early differentiation to mature stages^69^. Doublesex orthologs, including *Dmrt1*, exhibit male-biased expression in sea urchins, being highly expressed in the testis but absent in the ovaries^51^. Notably, *Dmrt1 is* also expressed at early stages of embryonic development, suggesting a potential role in early gonadal determination^39^. *FoxL2*, a highly conserved regulator of ovarian differentiation in chordates, even shows female-biased expression in mollusks^70,71^. In echinoderms, however, *FoxL2* is relatively abundantly expressed in both male and female gonads^72^, and single-cell RNA-seq analyses have revealed its presence in follicle cell–like clusters across three echinoderm species^73^. Interestingly, in sea urchins, *FoxL2* exhibits higher expression in undifferentiated gonads than in differentiated gonads, suggesting a potential role during early gonadal development in echinoderms. Further studies are needed to elucidate the functional roles of these genes in the process of gonadal differentiation in sea urchins.

To date, of the more than 20 identified master sex-determining genes in vertebrates, 13 are associated with the *TGF-β* signaling pathway, including *Amh*, *Amhr2*, *Bmpr1b*, *Gsdf*, and *Gdf6*^74^^.^ A total of 36 genes in the *TGF-β* signaling pathway were differentially expressed between differentiated and undifferentiated testes in sea urchin. Among them, thirty genes, such as *mothers against decapentaplegic homologs* (*Smad4* and *Smad1*), *fibrillin*, *bone morphogenetic protein* and its receptor, *multiple epidermal growth factor-like* (*EGF-like)* domains protein, and *mitogen-activated protein kinase* (*MAPK*) were highly expressed in undifferentiated testes, whereas six genes, such as *mothers against decapentaplegic homolog 6-like* (*Smad6-like*), *RING-box protein 1*(*Rbx1*), and *S-phase kinase-associated protein* (*SKP*), exhibited higher expression levels in differentiated testes. *Smad4* has been identified as a pivotal regulator of female germ cell viability, playing an indispensable role in oocyte differentiation and the progression of meiosis in murine models^75^. In contrast, the widespread expression of *Smad1* in the immature testis facilitates sufficient *BMP* signaling through the *Smad1* axis, thereby contributing to testicular development^76^. The high expression of *Smad6* protein at the onset of spermatogenesis suggests its potential involvement in testis differentiation^77^. *MAPK* signaling plays an established role in sex determination by being essential for the proper expression of *Sry* and *Sox9* during testis development^78^. *Rbx1* is highly expressed in the testis, particularly during the spermatocyte stage in male *Eriocheir sinensis*^79^, and exhibits dynamic distribution throughout oocyte maturation in mice, has been shown to be essential for gametogenesis, as its knockdown leads to metaphase arrest in most oocytes^80^, thereby implicating its crucial role in both spermatogenesis and oocyte meiotic progression. The members of *TGF-β* superfamily may play a crucial regulatory role in spermatogenesis and male gonadal differentiation in sea urchins.

The integrated transcriptome–metabolome analysis revealed that DEGs and DEMs were enriched in several key pathways, including retinol metabolism, amino acid metabolism, and steroid biosynthesis (Fig. 7). In the metabolomic analysis, both estradiol and testosterone were detected in gonads. However, their abundances did not differ significantly between differentiated and undifferentiated ovaries or testes. Although vertebrate-like sex steroid hormones have been detected in echinoderms using various methodologies^81,82^, many of the enzymes and receptors required for steroidogenesis in vertebrates, such as *CYP19a* which encodes aromatase responsible for converting androgens to estrogens, are absent in invertebrate genomes, and consequently, invertebrates, including echinoderms, are generally considered unable to synthesize sex steroids endogenously^83,84^. Therefore, the role of sex steroid hormones in sea urchin sex differentiation remains to be further investigated. Among the metabolites, retinoic acid (RA), linoleic acid, and arachidonic acid showed sex-specific patterns of accumulation and were strongly correlated with the expression of genes such as *CYP26*, *CYP1A*, and *CYP3A*, suggesting that retinoic acid signaling plays a central role in sea urchin gonadal differentiation (Fig. 7B, C), as it does in germ cell meiotic initiation in vertebrates. Retinoic acid has been shown to induce *Stra8* expression in embryonic ovaries, thereby influencing the initiation of meiosis in both female germ cells^85^. Meanwhile, the RA-degrading enzyme *Cyp26b1* prevents premature *Stra8* expression in embryonic testes, delaying meiosis until postnatal development^86^. *Cyp26a1* functions as a meiosis-inhibiting factor and plays a pivotal role in directing gonadal sexual fate toward the female pathway in zebrafish^87^. Moreover, retinoic acid signaling can promote ovarian development by activating female-specific genes such as *Foxl2*, whereas *DMRT1* restricts retinoic acid receptor (RARα) activity, thereby preventing *Foxl2* induction and safeguarding testicular fate^88^. In addition, the significant upregulation of ribosome and purine metabolism pathways in ovaries suggests that oogenesis may require a higher energy investment than spermatogenesis, indicating a potential trade-off between reproductive activity and somatic growth in sea urchins, as has been observed in other organisms^89,90^.

## Conclusions

In summary, we investigated gonadal development in *M. nudus* from 2 months post-fertilization (test diameter ~ 2.0 mm) to 2 years of age (test diameter ~ 40 mm), and found that gonadal differentiation begins around 2 years of age. Integrated transcriptomic and metabolomic analyses revealed that differentiated ovaries exhibited elevated amino acid and nucleic acid synthesis activity, sexual dimorphism in growth may exist among sea urchins. By combining multi-omics approaches, key genes such as *GATA4*, *HSD17B*, and *CYP17A1*, along with metabolites including retinoic acid, linoleic acid, and arachidonic acid, were identified as potential markers involved in sex differentiation and germ cell development. Among them, the retinol metabolism pathway appears to play a crucial role in regulating gonadal differentiation in sea urchins. Overall, this work enhances our understanding of the molecular regulation of gonadal differentiation in echinoderms and offers a valuable foundation for the development of sex control breeding strategies in *M. nudus* aquaculture.

## Materials and methods

### Experimental animals

Sea urchins used in the present study were laboratory-maintained individuals obtained from the Key Laboratory of Mariculture & Stock Enhancement in North China′s Sea, Ministry of Agriculture and Rural Affairs, Dalian Ocean University. They were maintained in 0.5 m^3^ seawater tanks under controlled conditions (17 ± 1 °C, salinity 30‰, pH 8.02 ± 0.04) and fed kelp once daily. Gonadal differentiation in juvenile sea urchins was first observed when individuals reached a test diameter of approximately 2 ± 0.2 mm. A total of 24 sea urchins were used for integrated transcriptomic and metabolomic analyses, as well as qRT-PCR validation, including 12 adults (test diameter ~ 40 mm) and 12 juveniles (test diameter ~ 20 mm). First, the genetic sex of sea urchins was determined by PCR amplification, as described in our laboratory’s previous study^41^. Subsequently, a subset of gonadal tissue was subjected to histological analysis to confirm the gonadal developmental stage. All adults were confirmed to be at the same reproductive stage (Figure S4).

### Histology, amino acid and fatty acid composition analysis in gonadal tissues

Gonadal tissues were collected by dissection from female and male sea urchins, respectively. A portion of each gonadal tissue was fixed in 4% paraformaldehyde (PFA) for histological analysis, while the remaining tissue was immediately flash-frozen in liquid nitrogen and stored at − 80 °C for subsequent RNA extraction, enzyme activity and amino acid composition analyses. Following fixation, the tissues were rinsed with phosphate-buffered saline (PBS) to remove excess PFA, then embedded in Optimal Cutting Temperature (O.C.T.) compound. Sections of 5 μm thickness were prepared using a Leica CM 1900 microtome, stained with hematoxylin and eosin (Servicebio, Wuhan, China), and examined for gonadal phases under a Leica DM4B microscope.

After genetic sex identification and histological examination of the gonads, three stage II ovaries and three stage II testes were selected for amino acid and fatty acid composition analysis. Amino acid profiles were analyzed using high-performance liquid chromatography (HPLC), and fatty acid composition was determined using gas chromatography–mass spectrometry (GC–MS). Both analyses were performed by Qingdao Kechuang Quality Testing Co., Ltd. (Qingdao, China) following standard protocols.

### RNA extraction and quantitative real-time PCR (qRT-PCR) analysis

Total RNA was separately extracted from three differentiated and three undifferentiated ovaries, as well as from three differentiated and three undifferentiated testes, for qRT-PCR analysis using the SV Total RNA Isolation System (Promega, Z3100), according to the manufacturer’s instructions. The RNA concentration and purity were measured with a NanoDrop 2000 spectrophotometer, and its integrity was evaluated via 1% agarose gel electrophoresis. Subsequently, 1 μg of high-quality RNA was reverse-transcribed into cDNA using the PrimeScript® First Strand cDNA Synthesis Kit (Takara, RR047A).

The qRT-PCR reaction was carried out on a LightCycler® 96 System (Roche, Mannheim, Germany) using the Hieff® qPCR SYBR Green Master Mix (Yeasen, Shanghai, China). Each 20 μL reaction mixture contained 10 μL of SYBR Green Mix, 6.4 μL of DEPC-treated water, 0.8 μL each of forward and reverse primers, and 2 μL of cDNA (1 μg/μL). The thermal cycling conditions were as follows: initial denaturation at 95 °C for 10 min, followed by 35 cycles of 95 °C for 15 s and 60 °C for 60 s. Primer sequences are listed in Table S7, all primers were confirmed to produce a single, specific amplification band (Figure S5). All analyses were performed with three biological replicates, and each reaction was conducted in triplicate. The *Ubiquitin* gene of *M. nudus* was used as the internal control to normalize the expression levels of target genes^37^. Relative gene expression levels were calculated using the 2^−ΔΔCT^ method. Statistical analysis was performed using an independent samples *t*-test in SPSS 22.0. A *p-value* < 0.05 was considered statistically significant.

### Transcriptomics analysis

Based on histological analysis of gonadal tissues, three samples each of differentiated ovaries (DO), differentiated testes (DT), undifferentiated ovaries (UDO), and undifferentiated testes (UDT) were used for RNA extraction and subsequent RNA sequencing (RNA-seq). Total RNA was extracted from the collected gonadal tissues using TRIzol reagent (Invitrogen, USA) according to the manufacturer’s protocol. Following quality assessment, 1 μg of total RNA per sample was used to construct sequencing libraries using the NEBNext® Ultra™ RNA Library Prep Kit for Illumina® (NEB, USA), according to the manufacturer’s protocol. The libraries were subsequently sequenced on the Illumina HiSeq 2000 platform to generate high-quality transcriptome data, provided by Biomarker Technologies Co., Ltd. (Qingdao, China). To ensure data quality, raw reads in FASTQ format were initially processed using in-house Perl scripts. Clean reads were obtained by removing adapter sequences, reads containing poly-N, and low-quality sequences. Quality metrics, including Q20, Q30, GC content, and sequence duplication levels, were calculated for the clean data. De novo transcriptome assembly was conducted using Trinity (version: trinity-v2.5.1)^91^. Gene function annotation was performed via BLAST searches (*E-value* < 1e^−5^) against major public databases, including NR (NCBI non-redundant protein sequences), Pfam, KOG, COG, eggNOG, Swiss-Prot, KEGG (Kyoto Encyclopedia of Genes and Genomes)^43^, and GO (Gene Ontology). Differential expression analysis was carried out using the DESeq R package (v1.10.1). *P-values* were adjusted using the Benjamini–Hochberg method to control the false discovery rate (FDR). Genes with adjusted *p*-values < 0.05 and |log₂ (fold change) |> 1 were considered significantly differentially expressed.

### Metabolite extraction and detection

A total of 24 gonadal tissue samples were collected, including six biological replicates each of DO, DT, UDO, and UDT, for non-targeted metabolomic profiling. Metabolomics analysis was conducted by Biomarker Technologies Co., Ltd. (Qingdao, China) using an LC–MS/MS platform. The analytical system consisted of a Waters ACQUITY UPLC I-Class PLUS system coupled to a Waters Xevo G2-XS QTof high-resolution mass spectrometer^92^. Chromatographic separation was performed using a Waters ACQUITY UPLC HSS T3 column (1.8 µm, 2.1 × 100 mm)^92^. Mass spectrometry was conducted in MSE mode with data acquisition controlled by MassLynx v4.2 software. Raw spectral data were processed using Progenesis QI software for peak detection, alignment, and normalization^92^. Metabolite identification was achieved through interrogation of the METLIN database and Biomarker’s in-house spectral library. Metabolites with missing values in more than 50% of samples per group were excluded, and internal standard normalization was applied to ensure comparability across samples^92^. Principal component analysis (PCA) and Spearman correlation analysis (SCA) were performed to assess sample reproducibility and data quality. Differential metabolites were identified using orthogonal partial least squares discriminant analysis (OPLS-DA) and univariate statistical tests (t-test)^93^, with significance thresholds set at fold change (FC) > *p* value < 0.05 and variable importance in projection (VIP) > 1. KEGG pathway enrichment analysis of differential metabolites was conducted using a hypergeometric distribution test^94^.

### Integrative analysis of metabolomics and transcriptomics

To explore coordinated molecular changes, DEGs and DEMs identified among the gonadal groups were subjected to integrative analysis. Prior to correlation analysis, gene expression and metabolite abundance data were standardized using Z-score transformation. Pearson correlation coefficients (PCC) were then calculated for each gene–metabolite pair. Pairs exhibiting strong correlations (|PCC|> 0.80, *p* < 0.05) were considered significantly associated. To further interpret these associations, shared KEGG metabolic pathways enriched by both DEGs and DEMs were identified. Correlation patterns within these common pathways were examined to uncover potential regulatory interactions and functional convergence between gene expression and metabolite accumulation.

## Ethics approval

The study was conducted in accordance with the ARRIVE guidelines and was approved by the Ethics Committee of Dalian Ocean University (Approval No. DLOU20250006).

## Human and animal rights

All personnel involved in the study underwent comprehensive training in animal care, handling techniques, and specific experimental procedures to ensure the minimization of animal discomfort and adherence to ethical standards.

## Supplementary Information

Below is the link to the electronic supplementary material.

Supplementary Material 1

Supplementary Material 2

Supplementary Material 3

Supplementary Material 4

Supplementary Material 5

Supplementary Material 6

Supplementary Material 7

Supplementary Material 8

Supplementary Material 9

Supplementary Material 10

Supplementary Material 11

Supplementary Material 12

Supplementary Material 13

Supplementary Material 14

## Data Availability

The datasets presented in this research are available from online repositories. Repository names and accession numbers are given below: NCBI’s BioProject data base: accession PRJNA1266096.

## References

[1] Stimpson, W. The Crustacea and Echinodermata of the pacific shores of North America. Houghton and Company. (1857).

[2] Adonin, L., Drozdov, A. & Barlev, N. A. Sea urchin as a universal model for studies of gene networks. *Front. Genet.* **11**, 627259 (2021). 33552139 10.3389/fgene.2020.627259 PMC7854572

[3] Marlétaz, F. et al. Analysis of the P. lividus sea urchin genome highlights contrasting trends of genomic and regulatory evolution in deuterostomes. *Cell Genomics.* **3**, 100295 (2023). 10.1016/j.xgen.2023.100295 PMC10112332 37082140

[4] Drozdov, A., Lebedev, E. & Adonin, L. Comparative analysis of bivalve and sea urchin genetics and development: Investigating the dichotomy in bilateria. *Int. J. Mol. Sci.* **24**, 17163 (2023). 38138992 10.3390/ijms242417163 PMC10742642

[5] Lawrence, J. M. Developments in aquaculture and fisheries science. *Edible Sea Urchin Biol Ecol.* **37**, 1–529 (2007).

[6] Takagi, S., Hasegawa, N., Watanabe, T., Sakai, Y. & Unuma, T. Dietary protein requirement for somatic growth and gonad production in the sea urchin *Strongylocentrotus* intermedius at different life stages. *Aquaculture* **575**, 739748 (2023).

[7] Liu, H., Chang, Y. Q. Sea urchin aquaculture in China. *Echinoderm aquaculture. *127–146 (2015).

[8] Yu, J., Yan, T., Kong, H. J. O. & Management, C. Exploring the management mode for breeding new species suitable for deep sea mariculture in China: Best practices, challenges, and prospects. *Ocean Coast. Manag.* **261**, 107508 (2025).

[9] Cen, Y. et al. The culture of the tropical sea urchin Salmacis sphaeroides: A new candidate for aquaculture in South China. *Aquac Rep.* **39**, 102371 (2024).

[10] Xu, W., Liu, Y., Li, M., Lu, S. & Chen, S. Advances in biotechnology and breeding innovations in China’s marine aquaculture. *Adv Biotechnol.* **2**, 38 (2024). 10.1007/s44307-024-00043-7 PMC11740861 39883290

[11] Rubilar, T. & Cardozo, D. Blue growth: Sea urchin sustainable aquaculture, innovative approaches. *Rev. Biol. Trop.* **69**, 474–486 (2021).

[12] Fisheries Bureau of the Ministry of Agriculture and Rural Affairs. China Agricultural Press, Beijing. (2025).

[13] Zilia, F., Orsi, L., Costantini, M., Tedesco, D. E. A. & Sugni, M. Case study of life cycle assessment and sustainable business model for sea urchin waste. *Clean Environ Syst.* **8**, 100108 (2023).

[14] Liu, L. et al. Relationships among morphological traits, body weight, and gonadal development in juvenile *Strongylocentrotus intermedius*. *Aquaculture* **537**, 736516 (2021).

[15] Archana, A. A. A. & Babu, K. Nutrient composition and antioxidant activity of gonads of sea urchin *Stomopneustes**variolaris*. *Food Chem.* **197**, 597–602 (2015). 26616993 10.1016/j.foodchem.2015.11.003

[16] Phillips, K. et al. Effect of gender, diet and storage time on the physical properties and sensory quality of sea urchin (*Evechinus chloroticus*) gonads. *Aquaculture* **288**, 205–215 (2009).

[17] Murata, Y., Yoshimura, H. & Unuma, T. Compositions of extractive components in the testes and ovaries of various sea urchins: Comparisons among species, sexes, and maturational status. *Fish. Sci.* **86**, 203–213 (2020).

[18] Zhao, C., Zhang, W., Chang, Y. & Liu, P. Test and gonad characteristics in different genders of cultivated sea urchins (*Strongylocentrotus intermedius*, *Agassiz*): First insight into sexual identification. *Afr. J. Biotech.* **9**, 7560–7563 (2010).

[19] Somjee, U., Shankar, A. & Falk, J. J. Can sex-specific metabolic rates provide insight into patterns of metabolic scaling?. *Integr. Comp. Biol.* **62**, 1460–1470 (2022). 10.1093/icb/icac135 35963649

[20] Chen, H. et al. Comparative physiological and transcriptomic profiling offers insight into the sexual dimorphism of hepatic metabolism in size-dimorphic spotted scat *(Scatophagus argus*). *Life.* **11**, 589 (2021). 34205643 10.3390/life11060589 PMC8233746

[21] Sun, J. L. et al. Sexual size dimorphism in golden pompano (*Trachinotus blochii*): Potential roles of changes in energy allocation and differences in muscle metabolism. *Front. Mar. Sci.* **9**, 1009896 (2022).

[22] Costa, D. N., Santosa, S. & Jensen, M. D. Sex differences in the metabolism of glucose and fatty acids by adipose tissue and skeletal muscle in humans. *Physiol. Rev.* **105**, 897–934 (2025). 39869194 10.1152/physrev.00008.2024 PMC12139471

[23] Pérez-Portela, R. & Leiva, C. Sex-specific transcriptomic differences in the immune cells of a key Atlantic-Mediterranean sea urchin. *Front. Mar. Sci.* **9**, 908387 (2022).

[24] Nagahama, Y., Chakraborty, T., Paul-Prasanth, B., Ohta, K., Nakamura, M. Sex determination, gonadal sex differentiation, and plasticity in vertebrate species. *Physiological reviews*. **101**, 1237–1308 (2021). 10.1152/physrev.00044.2019 33180655

[25] Curzon, A. Y., Shirak, A., Ron, M. & Seroussi, E. Master-key regulators of sex determination in fish and other vertebrates: A review. *Int. J. Mol. Sci.* **24**, 2468 (2023). 36768795 10.3390/ijms24032468 PMC9917144

[26] Song, H., Park, H. J., Lee, W. Y. & Lee, K. H. Models and molecular markers of spermatogonial stem cells in vertebrates: to find models in nonmammals. *Stem Cells Int.* **2022**(1), 4755514 (2022). 35685306 10.1155/2022/4755514 PMC9174007

[27] Song, Y., Hu, W., Ge, W. J. G. & Endocrinology, C. Establishment of transgenic zebrafish (*Danio rerio*) models expressing fluorescence proteins in the oocytes and somatic supporting cells. *Gen. Comp. Endocrinol.* **314**, 113907 (2021). 34543655 10.1016/j.ygcen.2021.113907

[28] Eno, C. C., Bottger, S. A. & Walker, C. W. Methods for karyotyping and for localization of developmentally relevant genes on the chromosomes of the purple sea urchin *Strongylocentrotus**purpuratus*. *Biol Bull.* **217**, 306–312 (2009). 20040754 10.1086/BBLv217n3p306

[29] Saotome, K., Kamimura, R., Kurokura, H. & Hirano, R. Male chromosomes of sea urchin hybrid andromerogones created with cryopreserved sperm. *Zoolog. Sci.* **19**, 185–189 (2002). 12012781 10.2108/zsj.19.185

[30] Eckelbarger, K. J. & Hodgson, A. N. Development, invertebrate oogenesis: A review and synthesis: comparative ovarian morphology, accessory cell function and the origins of yolk precursors. *Invertebr. Reprod. Dev.* **65**, 71–140 (2021).

[31] Hernandez, E., Vázquez, O. A., Torruco, A. & Rahman, M. S. Reproductive cycle and gonadal development of the Atlantic sea urchin Arbacia punctulata in the Gulf of Mexico: Changes in nutritive phagocytes in relation to gametogenesis. *Mar. Biol. Res.* **16**, 177–194 (2020).

[32] James, P., Siikavuopio, S., Johansson, G. S. A guide to the sea urchin reproductive cycle and staging sea urchin gonad samples. *Tromsø: Nofima*. (2011).

[33] Díaz-Martínez, J. P., Mejía-Gutiérrez, L. M., Islas-Villanueva, V. & Benítez-Villalobos, F. Trioecy is maintained as a time-stable mating system in the pink sea urchin *Toxopneustes* roseus from the Mexican Pacific. *Sci. Rep.* **12**, 21408 (2022). 36496463 10.1038/s41598-022-26059-4 PMC9741619

[34] Zhang, J. et al. Molecular cloning and sexually dimorphic expression analysis of nanos2 in the sea urchin, *Mesocentrotus nudus*. *Int. J. Mol. Sci.* **20**, 2705 (2019). 31159444 10.3390/ijms20112705 PMC6600436

[35] Jia, Z. et al. De novo transcriptome sequencing and comparative analysis to discover genes involved in ovarian maturity in *Strongylocentrotus nudus*. *Comp. Biochem. Physiol. D: Genomics Proteomics* **23**, 27–38 (2017). 28622611 10.1016/j.cbd.2017.05.002

[36] Gou, P., Wang, Z., Yang, J., Wang, X. & Qiu, X. Comparative transcriptome analysis of differentially expressed genes in the testis and ovary of sea urchin (*Strongylocentrotus intermedius*). *Fishes.* **7**, 152 (2022).

[37] Sun, Z. H., Zhang, J., Zhang, W. J. & Chang, Y. Q. Gonadal transcriptomic analysis and identification of candidate sex-related genes in *Mesocentrotus nudus*. *Gene* **698**, 72–81 (2019). 30825598 10.1016/j.gene.2019.02.054

[38] Mi, X. et al. Identification and profiling of sex-biased microRNAs from sea urchin *Strongylocentrotus nudus* gonad by Solexa deep sequencing. *Comp. Biochem. Physiol. D: Genomics Proteomics* **10**, 1–8 (2014). 24486540 10.1016/j.cbd.2014.01.001

[39] Pieplow, C., Furze, A., Gregory, P., Oulhen, N. & Wessel, G. M. Sex specific gene expression is present prior to metamorphosis in the sea urchin. *Dev. Biol.* **517**, 217–233 (2025). 39427857 10.1016/j.ydbio.2024.10.003

[40] Su, W.-Y. et al. Characterization and sexual dimorphic expression of Cytochrome P450 genes in gonads of the sea urchin (*Mesocentrotus nudus*). *Aquac Rep.* **36**, 102137 (2024).

[41] Cui, Z. et al. Identification of sex-specific markers through 2b-RAD sequencing in the sea urchin (*Mesocentrotus nudus*). *Front. Genet.* **12**, 717538 (2021). 34422019 10.3389/fgene.2021.717538 PMC8375557

[42] Kanehisa, M. Toward understanding the origin and evolution of cellular organisms. *Protein Sci.* **28**(11), 1947–1951 (2019). 31441146 10.1002/pro.3715 PMC6798127

[43] Kanehisa, M. & Goto, S. KEGG: Kyoto encyclopedia of genes and genomes. *Nucleic Acids Res.* **28**(1), 27–30 (2000). 10592173 10.1093/nar/28.1.27 PMC102409

[44] Kanehisa, M., Furumichi, M., Sato, Y., Matsuura, Y. & Ishiguro-Watanabe, M. KEGG: Biological systems database as a model of the real world. *Nucleic Acids Res.* **53**(D1), D672–D677 (2025). 39417505 10.1093/nar/gkae909 PMC11701520

[45] Hu, Y., Wang, B. & Du, H. A review on sox genes in fish. *Rev. Aquac.* **13**, 1986–2003 (2021).

[46] Chen, R. et al. Characterization and Functional analysis of the *17-beta hydroxysteroid dehydrogenase 2* (*hsd17b2*) gene during sex reversal in the ricefield Eel (*Monopterus albus*). *Int. J. Mol. Sci.* **25**, 9063 (2024). 39201749 10.3390/ijms25169063 PMC11354438

[47] Wang, Z. et al. Polymorphism within the GATA binding protein 4 gene is significantly associated with goat litter size. *Anim. Biotechnol.* **34**, 4291–4300 (2023). 36421983 10.1080/10495398.2022.2147533

[48] Yang, L. et al. *Cyp17a1* is required for female sex determination and male fertility by regulating sex steroid biosynthesis in fish. *Endocrinology* **162**, bqa205 (2021). 10.1210/endocr/bqab205 34581801

[49] Zhou, Z. et al. Function analysis and molecular characterization of *cyclin A* in ovary development of oriental river prawn Macrobrachium nipponense. *Gene* **788**, 145583 (2021). 33753150 10.1016/j.gene.2021.145583

[50] Zhang, W. et al. RNA interference analysis of the functions of *cyclin B* in male reproductive development of the oriental river prawn (*Macrobrachium nipponense*). *Genes* **13**, 2079 (2022). 36360319 10.3390/genes13112079 PMC9690022

[51] Cui, Z. et al. Testis-specific expression pattern of *dmrt1* and its putative regulatory region in the sea urchin (*Mesocentrotus nudus*). *Comp Biochem Physiol Part B: Biochem Mol Biol.* **257**, 110668 (2022). 10.1016/j.cbpb.2021.110668 34384887

[52] Sun, J.-J. et al. Identification and functional analysis of *foxl2* and *nodal* in sea cucumber *Apostichopus**japonicus*. *Gene Expr Patterns.* **44**, 119245 (2022). 35381371 10.1016/j.gep.2022.119245

[53] Heiat, M. et al. Knockdown of *SIX4* inhibits pancreatic cancer cells via apoptosis induction. *Med. Oncol.* **40**, 287 (2023). 37656231 10.1007/s12032-023-02163-x

[54] Richman, J. & Phelps, M. Activin signaling pathway specialization during embryonic and skeletal muscle development in rainbow trout (*Oncorhynchus mykiss*). *Mar. Biotechnol.* **26**, 766–775 (2024). 10.1007/s10126-024-10345-5 39052141

[55] Alshareef, A., Peters, A. C., Gélébart, P., Chen, W. & Lai, R. Gene methylation and silencing of *WIF1* is a frequent genetic abnormality in mantle cell lymphoma. *Int. J. Mol. Sci.* **22**, 893 (2021). 33477402 10.3390/ijms22020893 PMC7830226

[56] Walker, C. W., Unuma, T., Lesser, M. P. Gametogenesis and reproduction of sea urchins. *In Developments in aquaculture and fisheries science*. **37**, 11–33. (2007).

[57] Wang, H. et al. Metabolomic changes and polyunsaturated fatty acid biosynthesis during gonadal growth and development in the sea urchin *Strongylocentrotus intermedius*. *Comp Biochem Physiol Part D: Genom Proteom* **32**, 100611 (2019). 10.1016/j.cbd.2019.100611 31376663

[58] Wang, H., Ding, J., Ding, S. & Chang, Y. Integrated metabolomic and transcriptomic analyses identify critical genes in eicosapentaenoic acid biosynthesis and metabolism in the sea urchin *Strongylocentrotus intermedius*. *Sci. Rep.* **10**, 1697 (2020). 32015446 10.1038/s41598-020-58643-x PMC6997175

[59] Carré, G. A., Greenfield, A. The gonadal supporting cell lineage and mammalian sex determination: the differentiation of sertoli and granulosa cells. *Molecular Mechanisms of Cell Differentiation in Gonad Development*. **58**, 47–66 (2016). 10.1007/978-3-319-31973-5_3 27300175

[60] Walker, C. W., Lesser, M. & Unuma, T. Sea urchin gametogenesis: Structural, functional and molecular/genomic biology. *Dev Aquac fish Sci.* **38**, 25–43 (2013).

[61] Li, Z. & Yang, Y. Development, reproductive physiology and molecular mechanisms underlying testicular development and spermatogenesis in echinoderms: A marine invertebrate deuterostomes. *Mol. Reprod. Dev.* **92**, e70011 (2025). 39834110 10.1002/mrd.70011

[62] Walker, C. W., Harrington, L. M., Lesser, M. P. & Fagerberg, W. R. Nutritive phagocyte incubation chambers provide a structural and nutritive microenvironment for germ cells of *Strongylocentrotus* droebachiensis, the green sea urchin. *Biol. Bull.* **209**, 31–48 (2005). 16110092 10.2307/3593140

[63] Wei, H. et al. Sexual development of the hermaphroditic scallop Argopecten irradians revealed by morphological, endocrine and molecular analysis. *Front Cell Dev Biol.* **9**, 646754 (2021). 33796533 10.3389/fcell.2021.646754 PMC8007870

[64] LaVoie, H. A. The role of *GATA* in mammalian reproduction. *Exp. Biol. Med.* **228**, 1282–1290 (2003). 10.1177/153537020322801107 14681544

[65] Kyrönlahti, A. et al. *GATA4* regulates Sertoli cell function and fertility in adult male mice. *Mol. Cell. Endocrinol.* **333**, 85–95 (2011). 21172404 10.1016/j.mce.2010.12.019 PMC3026658

[66] Chen, M. et al. Transcriptomic analyses of hermaphroditic gonads at different stages revealing candidate genes for sex differentiation and gonad growth/maturation in QN *Orange scallops*. *Aquac. Res.* **53**, 3696–3705 (2022).

[67] Shangguan, X. et al. Cyp17a effected by endocrine disruptors and its function in gonadal development of Hyriopsis cumingii. *Gen. Comp. Endocrinol.* **323**, 114028 (2022). 35314150 10.1016/j.ygcen.2022.114028

[68] Kemiläinen, H. et al. The hydroxysteroid (17β) dehydrogenase family gene *HSD17B12* is involved in the prostaglandin synthesis pathway, the ovarian function, and regulation of fertility. *Endocrinology* **157**, 3719–3730 (2016). 27490311 10.1210/en.2016-1252

[69] Thitiphuree, T. Elucidation of biosynthetic pathway for sex steroids during gametogenesis in bivalve (Doctoral dissertation, Tohoku University). (2018).

[70] Biason-Lauber, A. WNT4, RSPO1, and FOXL2 in sex development. In seminars in reproductive medicine. In Seminars in reproductive medicine. Thieme medical publishers. **30**, 387–395 (2012) 10.1055/s-0032-1324722 23044875

[71] Ning, J. et al. Identification and functional analysis of a sex-biased transcriptional factor *Foxl2* in the bay scallop Argopecten irradians irradians. *Comp Biochem Physiol Part B: Biochem Mol Biol.* **256**, 110638 (2021). 10.1016/j.cbpb.2021.110638 34171478

[72] Zhang, J., Sun, Z.-H., Liu, B.-Z., Su, W.-Y. & Chang, Y.-Q. Sexually dimorphic expression of *foxl2* in the sea urchin (*Mesocentrotus nudus*). *Gene Expr. Patterns* **46**, 119280 (2022). 36202345 10.1016/j.gep.2022.119280

[73] Oulhen, N. et al. Conservation and contrast in cell states of echinoderm ovaries. *Mol. Reprod. Dev.* **91**, e23721 (2024). 38054259 10.1002/mrd.23721 PMC11153327

[74] Pan, Q. et al. Evolution of master sex determiners: TGF-β signalling pathways at regulatory crossroads. *Philos Trans R Soc B.* **376**, 20200091 (2021). 10.1098/rstb.2020.0091 PMC8273507 34247498

[75] Wu, Q. et al. Sexual fate change of XX germ cells caused by the deletion of *SMAD4* and *STRA8* independent of somatic sex reprogramming. *PLoS Biol.* **14**, e1002553 (2016). 27606421 10.1371/journal.pbio.1002553 PMC5015973

[76] Itman, C. & Loveland, K. L. Smads and cell fate: Distinct roles in specification, development, and tumorigenesis in the testis. *IUBMB Life* **65**, 85–97 (2013). 23300154 10.1002/iub.1115

[77] Itman, C. & Loveland, K. L. *SMAD* expression in the testis: An insight into BMP regulation of spermatogenesis. *Dev Dyn: Off Publ Am Assoc Anatomists.* **237**, 97–111 (2008). 10.1002/dvdy.21401 18069690

[78] Bogani, D. et al. Loss of mitogen-activated protein kinase kinase kinase 4 (MAP3K4) reveals a requirement for MAPK signalling in mouse sex determination. *PLoS Biol.* **7**, e1000196 (2009). 19753101 10.1371/journal.pbio.1000196 PMC2733150

[79] Zhang, J.-S., Li, X.-J., Yang, L., Li, W.-W. & Wang, Q. Expression pattern and functional analysis of the two RING box protein RBX in spermatogenesis of Chinese mitten crab Eriocheir sinensis. *Gene* **668**, 237–245 (2018). 29775751 10.1016/j.gene.2018.05.026

[80] Zhou, L. et al. The role of RING box protein 1 in mouse oocyte meiotic maturation. *PLoS ONE* **8**, e68964 (2013). 23874827 10.1371/journal.pone.0068964 PMC3708900

[81] Dewi, K. H., Masturah, M. & Wan Daud, W. R. Parameter optimization in the extraction of sea cucumber (*Holothuria scabra J*) as a source of testosterone. *Adv Mater Res.* **233**, 1358–1365 (2011).

[82] Thongbuakaew, T., Suwansa-Ard, S., Chaiyamoon, A., Cummins, S. F. & Sobhon, P. Sex steroids and steroidogenesis-related genes in the sea cucumber, *Holothuria**scabra* and their potential role in gonad maturation. *Sci. Rep.* **11**, 2194 (2021). 33500499 10.1038/s41598-021-81917-x PMC7838161

[83] Lafont, R. & Mathieu, M. Steroids in aquatic invertebrates. *Ecotoxicology* **16**, 109–130 (2007). 17238002 10.1007/s10646-006-0113-1

[84] Scott, A. P. Do mollusks use vertebrate sex steroids as reproductive hormones? II. Critical review of the evidence that steroids have biological effects. *Steroids* **78**, 268–281 (2013). 23219696 10.1016/j.steroids.2012.11.006

[85] Koubova, J. et al. Retinoic acid regulates sex-specific timing of meiotic initiation in mice. *Proc. Natl. Acad. Sci.* **103**, 2474–2479 (2006). 16461896 10.1073/pnas.0510813103 PMC1413806

[86] Kumar, S. et al. Sex-specific timing of meiotic initiation is regulated by *Cyp26b1* independent of retinoic acid signalling. *Nat. Commun.* **2**, 151 (2011). 21224842 10.1038/ncomms1136 PMC3034736

[87] Rodriguez-Mari, A. et al. Retinoic acid metabolic genes, meiosis, and gonadal sex differentiation in zebrafish. *PLoS ONE* **8**, e73951 (2013). 24040125 10.1371/journal.pone.0073951 PMC3769385

[88] Minkina, A. et al. *DMRT1* protects male gonadal cells from retinoid-dependent sexual transdifferentiation. *Dev. Cell* **29**, 511–520 (2014). 24856513 10.1016/j.devcel.2014.04.017 PMC4105363

[89] Powell, M. L., Marsh, A. G. & Watts, S. A. Biochemical and energy requirements of gonad development in regular sea urchins. *Dev Aquac Fish Sci.* **43**, 51–64 (2020).

[90] Marsh, A. G., Powell, M. L. & Watts, S. A. Biochemical and energy requirements of gonad development. *Dev Aquac Fish Sci.* **38**, 45–57 (2013).

[91] Grabherr, M. G. et al. Full-length transcriptome assembly from RNA-Seq data without a reference genome. *Nat. Biotechnol.* **29**, 644–652 (2011). 21572440 10.1038/nbt.1883 PMC3571712

[92] Wang, J. et al. Serum metabolomics for early diagnosis of esophageal squamous cell carcinoma by UHPLC-QTOF/MS. *Metabolomics* **12**, 116 (2016).

[93] Thévenot, E. A., Roux, A., Xu, Y., Ezan, E. & Junot, C. J. Analysis of the human adult urinary metabolome variations with age, body mass index, and gender by implementing a comprehensive workflow for univariate and OPLS statistical analyses. *J. Proteome Res.* **14**, 3322–3335 (2015). 26088811 10.1021/acs.jproteome.5b00354

[94] Yu, G., Wang, L.-G., Han, Y. & He, Q.-Y. clusterProfiler: An R package for comparing biological themes among gene clusters. *Omics: J Integr Biol.* **16**, 284–287 (2012). 10.1089/omi.2011.0118 PMC3339379 22455463

