# Supplementary material for: Integrated application of transcriptomics and metabolomics provides insights into gonadal differentiation in Mesocentrotus nudus
Source: Sci Rep. 2025 Dec 20;16:2715. doi: 10.1038/s41598-025-32582-x (PMC12824366; doi:10.1038/s41598-025-32582-x)
Supplement: Supplementary file 6 — Supplementary Material 6 [file 41598_2025_32582_MOESM6_ESM.docx]

Table S1 Fatty acid composition of sea urchin *Mesocentrotus nudus* gonads.

| Fatty acid | % Composition in ovary | % Composition in testis |
| --- | --- | --- |
| SFA |  |  |
| C12:0 | 0.04±0.00 | 0.05±0.01 |
| C13:0 | 0.05±0.02 | 0.07±0.01 |
| C14:0 | 11.55±1.17 | 11.68±1.19 |
| C15:0 | 0.69±0.10 | 0.63±0.13 |
| C16:0 | 24.2±1.13 | 22.70±1.26 |
| C17:0 | 0.26±0.02 | 0.30±0.08 |
| C21:0 | 11.66±0.43 | 10.15±0.70 |
| C20:0 | 1.29±0.09 | 1.22±0.04 |
| C18:0 | 3.12±0.15 | 3.68±0.18 |
| MUFA |  |  |
| C14:1 | 0.40±0.02 | 0.54±0.09 |
| C16:1 | 1.92±0.21 | 2.02±0.16 |
| C20:1 | 5.67±0.32 | 5.36±0.11 |
| C22:1n9 | 5.11±0.15 | 6.01±0.56 |
| C18:1n9c | 1.42±0.12 | 1.69±0.17 |
| PUFA |  |  |
| ω3 |  |  |
| C18:3n3 | 2.08±0.30 | 1.72±0.19 |
| C20:3n3 | 4.66±0.72 | 3.73±0.66 |
| C22:6n3 | 1.69±0.56 | 1.13±0.88 |
| C20:5n3 | 13.5±1.16 | 13.13±1.10 |
| ω6 |  |  |
| C20:4n6 | 7.54±1.80 | 10.12±0.50 |
| C20:3n6 | 0.86±0.06 | 1.12±0.07 |
| C18:3n6 | 0.23±0.05 | 0.30±0.04 |
| C22:2 | 0.20±0.06 | 0.17±0.17 |
| C20:2 | 1.32±0.12 | 1.71±0.28 |
| C18:2n6c | 0.54±0.06 | 0.77±0.77 |
| Σ SFA | 52.86 | 50.48 |
| Σ MUFA | 14.52 | 15.62 |
| Σ PUFA | 32.62 | 33.9 |
| Σ ω3 | 21.93 | 19.71 |
| Σ ω6 | 10.69 | 14.19 |
| ω6: ω3 | 0.48 | 0.72 |

SFA: Saturated Fatty Acids, MUFA: Monounsaturated Fatty Acids, ω-6 PUFA: ω-6 Polyunsaturated Fatty Acids, ω-3 PUFA: ω-3 Polyunsaturated Fatty Acids.
