# Supplementary material for: Integrated application of transcriptomics and metabolomics provides insights into gonadal differentiation in Mesocentrotus nudus
Source: Sci Rep. 2025 Dec 20;16:2715. doi: 10.1038/s41598-025-32582-x (PMC12824366; doi:10.1038/s41598-025-32582-x)
Supplement: Supplementary file 7 — Supplementary Material 7 [file 41598_2025_32582_MOESM7_ESM.docx]

Table S2 Summary statistics of RNA-Seq dates

| Sample name | Read number | Base number | GC content | % ≥ Q30 |
| --- | --- | --- | --- | --- |
| DO-1 | 24,142,785 | 7,198,396,646 | 43.27% | 95.73% |
| DO-2 | 23,807,079 | 7,096,536,416 | 43.50% | 96.02% |
| DO-3 | 23,133,336 | 6,902,517,430 | 43.65% | 95.50% |
| DT-1 | 25,331,724 | 7,547,817,780 | 41.34% | 95.68% |
| DT-2 | 21,200,915 | 6,331,358,974 | 42.41% | 96.13% |
| DT-3 | 20,809,339 | 6,211,362,648 | 41.61% | 95.49% |
| UDO-1 | 23,143,357 | 6,910,645,030 | 44.12% | 95.57% |
| UDO-2 | 22,351,215 | 6,677,579,556 | 44.14% | 95.38% |
| UDO-3 | 25,104,072 | 7,488,867,894 | 43.48% | 95.73% |
| UDT-1 | 22,801,625 | 6,804,547,794 | 43.42% | 95.61% |
| UDT-2 | 20,957,321 | 6,255,161,600 | 44.21% | 95.90% |
| UDT-3 | 25,077,718 | 7,486,846,322 | 43.22% | 95.25% |

DO, differentiated ovaries; DT, differentiated testes; UDO, undifferentiated ovaries; UDT, undifferentiated testes.
