# Supplementary material for: Integrated application of transcriptomics and metabolomics provides insights into gonadal differentiation in Mesocentrotus nudus
Source: Sci Rep. 2025 Dec 20;16:2715. doi: 10.1038/s41598-025-32582-x (PMC12824366; doi:10.1038/s41598-025-32582-x)
Supplement: Supplementary file 8 — Supplementary Material 8 [file 41598_2025_32582_MOESM8_ESM.docx]

Table S3 Summary statistics of assembly.

| Length range | Transcript | Unigene |
| --- | --- | --- |
| 200~300 | 104,460（25.21%） | 0（0%） |
| 300~500 | 74,366（17.95%） | 14,633（25.78%） |
| 500~1000 | 80,076（19.33%） | 9,397（16.55%） |
| 1000~2000 | 76,620（18.49%） | 15,368（27.07%） |
| 2000 + | 78,781（19.02%） | 17,372（30.60%） |
| Total number | 414,305 | 56,770 |
| Total length | 505,734,434 | 100,697,795 |
| N50 length | 2,315 | 2,846 |
| Mean length | 1220.68 | 1773.79 |
