# Supplementary material for: Integrated application of transcriptomics and metabolomics provides insights into gonadal differentiation in Mesocentrotus nudus
Source: Sci Rep. 2025 Dec 20;16:2715. doi: 10.1038/s41598-025-32582-x (PMC12824366; doi:10.1038/s41598-025-32582-x)
Supplement: Supplementary file 9 — Supplementary Material 9 [file 41598_2025_32582_MOESM9_ESM.docx]

Table S4 Annotation of unigenes functional

| Annotated databases | Annotated number | 300 ≤ length < 1000 | Length ≥ 1000 |
| --- | --- | --- | --- |
| COG_Annotation | 5655 | 565 | 5090 |
| GO_Annotation | 16056 | 2091 | 13944 |
| KEGG_Annotation | 18100 | 2517 | 15583 |
| KOG_Annotation | 13133 | 1383 | 11750 |
| Pfam_Annotation | 18058 | 2092 | 15966 |
| Swissprot_Annotation | 15789 | 1806 | 13983 |
| TrEMBL_Annotation | 24302 | 4292 | 20010 |
| eggNOG_Annotation | 19227 | 3197 | 16030 |
| NR_Annotation | 24306 | 4306 | 20000 |
| All_Annotated | 25652 | 4771 | 20860 |
