# Supplementary material for: Integrated application of transcriptomics and metabolomics provides insights into gonadal differentiation in Mesocentrotus nudus
Source: Sci Rep. 2025 Dec 20;16:2715. doi: 10.1038/s41598-025-32582-x (PMC12824366; doi:10.1038/s41598-025-32582-x)
Supplement: Supplementary file 12 — Supplementary Material 12 [file 41598_2025_32582_MOESM12_ESM.docx]

Table S7. The primers sequences used for qRT-PCR analysis on selected DEGs

| Gene name | Forward primer (5’-3’) | Reverse primer(5’-3’) | Length  (bp) |
| --- | --- | --- | --- |
| *SOX14* | GCTATCACCAGAGAAGATGGACACCAG | GATTGCTGTGCTCCTCTGCCTCTG | 249 |
| *HSD17B* | GTCGTGCAACCTGCCAGCTATTCG | GGTAGCAGGCTTCTTGAAGGTATCCG | 209 |
| *GATA4* | GTCACACCACCATTGGTATTAG | GTGAGCAAACCCTACATCTTAC | 118 |
| *CYP17A1* | GATGATTATGTGCTCAGGTGTG | CATACAAAGCAGGAAGGGTTAG | 119 |
| *CYCLIN A* | GACAATACCAGCAGAAATGGACCAC | CGAATGGCTTGCCCTGTTGTATCC | 187 |
| *CYCLIN B* | CTGATGCTCATGAACCTATTGCTGC | CTCTATTAGGTTCCGAGGACAACTGG | 181 |
| *Ubiquitin* | TGGTCGCACTCTCTCAGACTACAAC | TGCCGTCTCTCAATTTGTCTATAGC | 232 |
