# Supplementary material for: Integrated application of transcriptomics and metabolomics provides insights into gonadal differentiation in Mesocentrotus nudus
Source: Sci Rep. 2025 Dec 20;16:2715. doi: 10.1038/s41598-025-32582-x (PMC12824366; doi:10.1038/s41598-025-32582-x)
Supplement: Supplementary file 13 — Supplementary Material 13 [file 41598_2025_32582_MOESM13_ESM.docx]

**Supplementary Information**

Supplementary Figure S1. Principal component analysis. DO, differentiated ovaries; DT, differentiated testes; UDO, undifferentiated ovaries; UDT, undifferentiated testes. X, Y, Z stands for three principal components. values in brackets are contributions of Principal component.

Supplementary Figure S2. The volcano plot displays the differentially expressed genes between the compared gonadal groups. Each dot represents a gene, with red and green dots indicate significantly upregulated and downregulated genes, respectively. Black dots represent genes with non-significant expression changes.

Supplementary Figure S3. The Venn diagram of differential metabolites between the compared gonadal groups. The numbers in the overlapping areas indicate the shared differential metabolites between groups, while the numbers in the non-overlapping areas indicate the unique differential metabolites for each group. DO, differentiated ovaries; DT, differentiated testes; UDO, undifferentiated ovaries; UDT, undifferentiated testes.

Supplementary Figure S4. Gonads histological analysis of M. nudus. ovaries (A–F) and testes (G–L) during stages 2; undifferentiated ovaries (M–R); Undifferentiated testes (S–Z). Bar = 50 µm.

Supplementary Figure S5. Validations of primers used for qRT-PCR.

Supplementary Table S1. Fatty acid composition of sea urchin Mesocentrotus nudus gonads.

Supplementary Table S2. Summary statistics of RNA-Seq data.

Supplementary Table S3. Summary statistics of assembly.

Supplementary Table S4. Annotation of unigenes functional.

Supplementary Table S5. Raw FPKM values of sex-biased genes between differentiated and undifferentiated gonads of M. nudus.

Supplementary Table S6. Correlation between the metabolomics and transcriptomics of M. nudus

Supplementary Table S7. The primers sequences used for qRT-PCR analysis on selected DEGs
