# Supplementary material for: Integrated application of transcriptomics and metabolomics provides insights into gonadal differentiation in Mesocentrotus nudus
Source: Sci Rep. 2025 Dec 20;16:2715. doi: 10.1038/s41598-025-32582-x (PMC12824366; doi:10.1038/s41598-025-32582-x)
Supplement: Supplementary file 14 — Supplementary Material 14 [file 41598_2025_32582_MOESM14_ESM.pdf]

KEGG Database Project
Kanehisa Laboratories
www.kanehisa.jp

Ref: 254199

Permission is granted to Scientific Reports of Springer Nature Ltd to publish both in print and digital under the CC BY 4.0 open access license the result of using KEGG and the following KEGG images in the article "Integrated application of transcriptomics and metabolomics provides insights into gonadal differentiation in Mesocentrotus nudus" written by Zhihui Sun and colleagues:

- ECM-receptor interaction (map04512)
- TGF-beta signaling pathway (map04350)
- Proteasome (map03050)
- Homologous recombination (map03440)
- Purine metabolism (map00230)
- DNA replication (map03030)
- Mismatch repair (map03430)
- Pyruvate metabolism (map00620)
- Fatty acid metabolism (map01212)
- Aminoacyl-tRNA biosynthesis (map00970)
- Steroid biosynthesis (map00100)
- Carbon metabolism (map01200)
- Glycolysis / Gluconeogenesis (map00010)
- Amino sugar and nucleotide sugar metabolism (map00520)
- Valine, leucine and isoleucine degradation (map00280)
- Fatty acid elongation (map00062)
- Biosynthesis of amino acids (map01230)
- Arginine and proline metabolism (map00330)
- Phenylalanine, tyrosine and tryptophan biosynthesis (map00400)
- Citrate cycle (TCA cycle) (map00020)
- Propanoate metabolism (map00640)
- Biosynthesis of unsaturated fatty acids (map01040)
- Terpenoid backbone biosynthesis (map00900)
- Proteasome (map03050)
- 2-Oxocarboxylic acid metabolism (map01210)
- Oxidative phosphorylation (map00190)
- Cysteine and methionine metabolism (map00270)
- Longevity regulating pathway - worm (map04212)
- Chloroalkane and chloroalkene degradation (map00625)
- Biosynthesis of various antibiotics (map00998)
- Methane metabolism (map00680)
- Inositol phosphate metabolism (map00562)
- Histamine H2/H3 receptor agonists/antagonists (map07227)
- Biosynthesis of alkaloids derived from shikimate pathway (map01063)
- Biosynthesis of various other secondary metabolites (map00997)
- Serotonergic synapse (map04726)
- Methane metabolism (map00680)
- Isoquinoline alkaloid biosynthesis (map00950)
- Phenylalanine, tyrosine and tryptophan biosynthesis (map00400)
- Butanoate metabolism (map00650)
- Terpenoid backbone biosynthesis (map00900)
- Biosynthesis of plant hormones (map01070)
- Biosynthesis of siderophore group nonribosomal peptides (map01053)
- Eicosanoids (map07034)

subject to the condition that the original source is acknowledged by citing at least one KEGG paper.

Permission granted: Yixuan Song

Yixuan Song, Kanehisa Laboratories

Date: 25 November 2025

Copyright holder: Kanehisa Laboratories
